# Accelerometer-Assessed Physical Activity and Cognitive Performance among European Adults Aged 50+: The Mediating Effects of Social Contacts and Depressive Symptoms

**DOI:** 10.3390/healthcare10112279

**Published:** 2022-11-14

**Authors:** Ella Cohn-Schwartz, Rabia Khalaila

**Affiliations:** 1 Epidemiology, Biostatistics, and Community Health Sciences, Faculty of Health Sciences, Ben-Gurion University, Beer-Sheva 8410501, Israel; 2 Zefat Academic College, 11 Jerusalem St., P.O.B. 169, Zefat 13206, Israel; 3 Global Brain Health Institute, University of California, San-Francisco, CA 94107, USA

**Keywords:** accelerometer-based physical activity, intensity gradient, depression, social contacts, cognitive health, adults

## Abstract

Background: Cognitive decline is a major public health concern worldwide and it is vital to identify and better understand effective population-based means to improve cognitive performance in old age. The current study set out to examine the links between accelerometer-based physical activity with cognitive performance in later life, as well the indirect pathways through one’s social network contacts and depression. Method: We used data from 855 participants aged 50 and above who took part in a cross-sectional accelerometer study as part of the Survey of Ageing, Retirement and Health (SHARE). Cognitive function was measured as an average score of fluency, immediate and delayed recall tests, social contacts were the average contact frequency with members of the social support network, and depression was the Euro-D summary score of depressive symptoms. A multiple mediation analysis was conducted to test the direct and indirect associations between total physical activity (intensity gradient) and cognitive function, as well as the mediation of this association by social contacts and depressive symptoms. Results: Intensity of physical activity was directly related to better cognitive performance (*B* = 0.170, *p* = 0.007). The association was partially mediated by social contacts (*B* = 0.022, 95% CI 0.005, 0.046) and depressive symptoms (*B* = 0.009, 95% CI 0.009, 0.025), such that total physical activity was linked to cognitive health via more frequent contacts with network members and low depressive symptoms. Conclusions: Practitioners might consider encouraging a physically active lifestyle that involves social interactions to support better cognitive aging and mental health.

## 1. Introduction

One of the greatest concerns in later life is of poor cognitive performance. Furthermore, cognitive decline is a major public health concern around the world as a result of global increased life expectancy and population aging [1]. People with cognitive decline face an increased risk of progressing to mild cognitive impairment (MCI) and developing dementia [2,3]. Worse cognitive function is associated with loss of independence, low quality of life, reduced functional capacity, higher healthcare costs, and mortality [4,5]. Therefore, it is vital to identify effective population-based means to improve cognitive performance in later life. 

Physical activity (PA) is any bodily movement that increases energy expenditure [6]. It is a modifiable health behavior that is associated with better cognitive performance [7,8,9,10,11]. However, most of the relevant literature is based on self-reported PA, a method which suffers from various limitations; a systematic review of self-reported physical activity measures in older adults noted that most self-report measures have low validity due to issues such as over-reporting and under-reporting. Recall bias is an additional limitation since PA engagement of older adults is often intermittent, sporadic, or unstructured and thus difficult to remember accurately [12]. 

The ability to measure PA objectively, using accelerometers, provides an opportunity to better understand the role of PA in relation to cognitive performance in later life. Studies on objectively assessed physical activity and global cognitive function generally indicate that greater duration of PA and high intensity PA are beneficial for global cognitive function [13,14,15]. Such studies indicated, for example, that the number of accelerometer-assessed daily steps were associated with a reduced subjective cognitive decline rate after two years among Chinese older adults [2]. Higher light and moderate-to-vigorous accelerometer-assessed physical activity was associated with a reduced rate of decline in cognitive ability two years later among Chinese older adults [16], although a different study, conducted among American adults, found that only moderate-to-vigorous accelerometer-assessed physical activity was related to better performance in memory and executive function [17]. 

However, the output variables derived from accelerometer data are commonly limited to overall activity level (average acceleration) and time spent in specific intensity categories such as moderate-to-vigorous physical activity [14,16,17]. The intensity gradient is a novel metric that captures the intensity distribution of the physical activity profile [18]. It differs from other accelerometer-based measures by reflecting the distribution of the intensity of activities over a whole day. Major strengths of the intensity index are that it covers the entire 24 h period and the full intensity spectrum, allowing the acquisition of all the physical activity performed. It also does not impose arbitrary cut points on data, thus overcoming the lack of comparability between studies due to the wide range of cut points used, and is more sensitive to subtle differences in physical activity [19]. The intensity gradient has been associated with better physical health and health-related quality of life; for example, previous studies found the intensity gradient to be negatively associated with BMI, waist-to-height ratio, metabolic syndrome, and percent of body fat in children and adults [19,20,21], even more than the average acceleration [21,22]. Given the independent positive associations between the intensity gradient and physical function, it has been suggested that the intensity distribution of the physical activity profile may be of particular relevance in later life [18], even though its association with cognition has not yet been examined. Therefore, our study will add to the literature by investigating the intensity gradient of accelerometer-measured PA in relation to cognitive performance in later life. 

An additional issue that requires better understanding is the identification of the mediators of the association between PA and cognitive performance [16,23]. One such potential mediator is through more frequent social interactions—physical activity might involve social interactions, which can be related to better cognition. The cognitive enrichment hypothesis maintains that a cognitively stimulating environment is beneficial for better cognitive aging [24]. In accordance with that hypothesis, the “use it or lose it” hypothesis suggests that social interactions might provide such a stimulating environment and provide “cognitive exercise” which stimulates the mind and preserves cognitive functions [24,25]. Several studies indicate that persons who report frequent social interactions also function better cognitively [26,27,28,29]. However, these findings are not consistent [30,31,32]. 

An additional potential explanatory factor is depressive symptoms, such that adults who are more physically active also enjoy better mental health, which in turn improves their cognitive performance. Depressive symptoms are especially meaningful to measure as depression can increase in the context of the major challenges of the 21st century [33]. Accordingly, PA has been linked with higher levels of depressive symptoms, although this was mostly examined via self-reported PA [34,35,36,37]. Additionally, higher levels of depressive symptoms have been found to be negatively related to cognitive performance [11,24,29,38,39].

Thus, the current study will examine the association of PA as indicated by the accelerometer-measured intensity gradient with cognitive performance, while also investigating the potential mediators of depressive symptoms and social contacts. Our study is novel in examining the intensity gradient in relation to cognition during later life, and also in our examination of the mediators of this association. We address three hypotheses:

**H1**: *Accelerometer-assessed PA is positively linked with cognitive performance*.

**H2**: *Accelerometer-assessed physical activity is also indirectly linked with cognitive performance through contact frequency with the social network. That is, PA will be positively associated with contact frequency with the members of one’s social network, which will be related to better cognitive performance in later life*.

**H3**: *Accelerometer-assessed PA is also indirectly linked with cognitive performance through depressive symptoms. That is, PA will be negatively associated with depressive symptoms, which will be related to worse cognitive performance*.

## 2. Methods

We used data from the eighth wave of data collected by the Survey of Health, Ageing and Retirement in Europe (SHARE) [40]. SHARE is a cross-national panel survey of European community-dwelling adults aged 50 and above and their partners of any age. Data is collected via professional survey agencies in each participating country using face-to-face interviews. Participants are sampled to be representative of the population of adults aged 50 and above [41]. There are indications that cognitive aging, i.e., a decline in cognitive function, is present in adults in their 50s [42]. This data collection wave of SHARE was innovative in its use of accelerometers to collect physical activity data in a subset of ten participating countries to ensure geographic variation: Belgium, Czech Republic, Denmark, France, Germany, Italy, Poland, Slovenia, Spain, and Sweden. The study sample was determined such that for each of the countries, the defined target sample was a net sample of 200 participants. To obtain complete data from 200 respondents, a gross sample of about 710 individuals per country was needed, which took into account that not all sampled participants will consent to participate, wear, and return the device or have complete information from eight days. The gross sample was selected from each country’s longitudinal sample and excluded younger partners aged under 50. The sample was stratified by age group and activity level that was self-reported in the previous wave. Sample members were selected in proportion to the size of the strata in the Wave 7 panel sample by country but with a fixed minimum number of 50 respondents per stratum. The minimum size for strata was chosen because some strata were very small, especially those of respondents with low self-reported physical activity. The fixed minimum resulted in an intended oversample of this group. According to this sampling method, 855 participants were recruited for the accelerometer gross subsample and they make up the current study sample (for additional information please see: [37]). 

Respondents in the accelerometer subsample were asked to wear the accelerometer (Axivity AX3) for eight consecutive days (day and night). Participating respondents received the accelerometer after their face-to-face regular interview via the post. The participants wore the accelerometer on their upper thigh by attaching it using a medical-style adhesive tape. The data was collected between October 2019–March 2020 until data collection was stopped due to the outbreak of the COVID-19 pandemic [43]. 

The SHARE study is subject to continuous ethics review. Wave 8 was reviewed and approved by the Ethics Council of the Max Planck Society. In addition, the country implementations of SHARE were reviewed and approved by the respective ethics committees or institutional review boards whenever this was required. 

## 3. Measures

### 3.1. Dependent Variable

We measured cognitive performance by combining three cognitive tests into one measure. The cognitive tests included immediate recall, delayed recall, and verbal fluency and are validated measurements of cognition in later life [29,44]. The tasks to test the immediate and delayed recall are episodic memory tasks that assess abilities for short-term verbal learning, memory, and information retention [45]. In the short-term memory task, the interviewer reads aloud ten words and asks the participant to repeat as many words as possible. Five–ten minutes later, the participant is asked to repeat these words again, for the delayed memory task. The final score for each task ranges from 0 to 10 points. The verbal fluency task assesses executive functioning and language abilities [46]. In this task, the participant is asked to name as many animals as possible within one minute. Participants who answered more than 45 names per minute received a final score of 45. The final measure of cognitive health calculated the average of the standardized scores of these three factors. We performed factor analysis for the three variables and saw that they indeed constitute one single factor, and can thus be used as a single average score. 

### 3.2. Independent Variable

In the current study, we used the intensity gradient (IG) metric. Accelerometer-assessed physical activity is an analytical measure of overall physical activity during the day that reflects the volume and intensity of a 24-h activity profile [20]. SHARE provides a dataset with various accelerometer-based measures that were generated with the R-package GGIR version 2.2-0 [47], running with R version 4-0-3 [48], including the intensity gradient metric. The intensity gradient reflects the distribution of intensity of activities carried out within a span of 24 h. It describes the negative curvilinear relationship between activity intensity and the time accumulated at that intensity. All measures calculated with GGIR are based on the Euclidian norm minus one with negative values set to zero (ENMO) [49,50]. The intensity gradient is always negative, reflecting the drop in time accumulated as intensity increases; a more negative (lower) gradient reflects a steeper drop with little time accumulated at mid-range and higher intensities, while a less negative (higher) gradient reflects a shallower drop with more time spread across the intensity range. Higher values indicate that a greater proportion of the activity was spent at high intensity during the day. A lower measure indicates that the participant spent less time in intense activity during the day [18]. The measurement of physical activity using the intensity gradient is now common and validated [20,47,49,50,51]. Participants were excluded if they failed calibration and if they had fewer than 3 days of valid wear (defined as >16 h/d). These exclusion criteria were established by Rowlands et al. [18] in their development of the intensity gradient and used in additional studies with the intensity gradient [21,22].

### 3.3. Mediators

Contact frequency with the social network was evaluated through the participant’s subjective assessment of the frequency of contact with members of their social network, as indicated in the name-generator task. This task has been validated and used in various surveys of adults in later life (see, for example: [52,53,54]). Each participant was asked to name up to seven people with whom they talk about important matters [29]. The contact frequency with each member in the social network was assessed using one question with a 7-point response scale ranging from “daily” to “never”, which was re-coded before calculating the final score. The final score calculated the average contact frequency with network members, such that a high score on this measure indicated a higher frequency of contact with the social network members [29].

Depressive symptoms were measured using the EURO-D scale [55]. The index includes 12 symptoms of depression: depressed mood, concentration, enjoyment, guilt, sleep, interest, irritability, fatigue, pessimism, suicidal tendencies, appetite, and tearfulness. Each participant was asked to indicate for each symptom whether it was present (1) or not present (0) in the last month. The final score summed the answers with a range of 0 to 12, and a greater score indicated more depressive symptoms. The scale has been validated previously [56] and it has good reliability in the current study (α = 0.71). 

### 3.4. Covariates 

We used various background measures as covariates in the multivariate analyses of this study. These variables included demographic characteristics of gender (male/female) and age, which was measured as a continuous scale. Education was measured using the ISCED-1997 classification that includes 7 education levels (from 0 = none to 6 = second stage of tertiary education). Education was categorized into two levels: 0 = upper secondary education and below or 1 = post-secondary education. The network size index included the total number of people in the network with whom the participant talks to about important matters. Self-reported physical activity was measured by two questions about the participant’s engaging in moderate (“sports and activities that require a moderate level of energy”) and vigorous activity (“sports or activities that are vigorous”). These measures were combined into one measure in the current analysis insofar as about 80% of the respondents reporting vigorous activity also reported doing moderate activity. The combined measure thus reflected two levels: 0 = never vigorous nor moderate physical activity, 1 = other (vigorous or moderate physically active) [57,58,59]. In addition, the current study included health-related factors as covariates. These factors include three measures: self-rated health, number of chronic diseases, and health-related activity limitations. For self-rated health, each participant was asked to rate their health in the past year on a 5-point scale including (1 = excellent to 5 = poor). This was dichotomized to 1 = very good/excellent or 0 = less than very good [60,61]. The number of chronic diseases were measured by asking the participant to select (1 = yes, 0 = no) if they had ever been diagnosed with each disease from list of 14 diseases such as hypertension, heart attack, diabetes, etc. [62,63]. The third health measure was the Global Activity Limitation Indicator (GALI), which evaluates one’s activity limitation due to his or her physical health in the last six months. It was a single item with a 3-point response scale: severely limited, limited but not severely, and not limited at all. This indicator was dichotomized as 0 = not having any limitations and 1 = severe and moderate limitations [64]. 

## 4. Data Analyses

**Figure 1:**
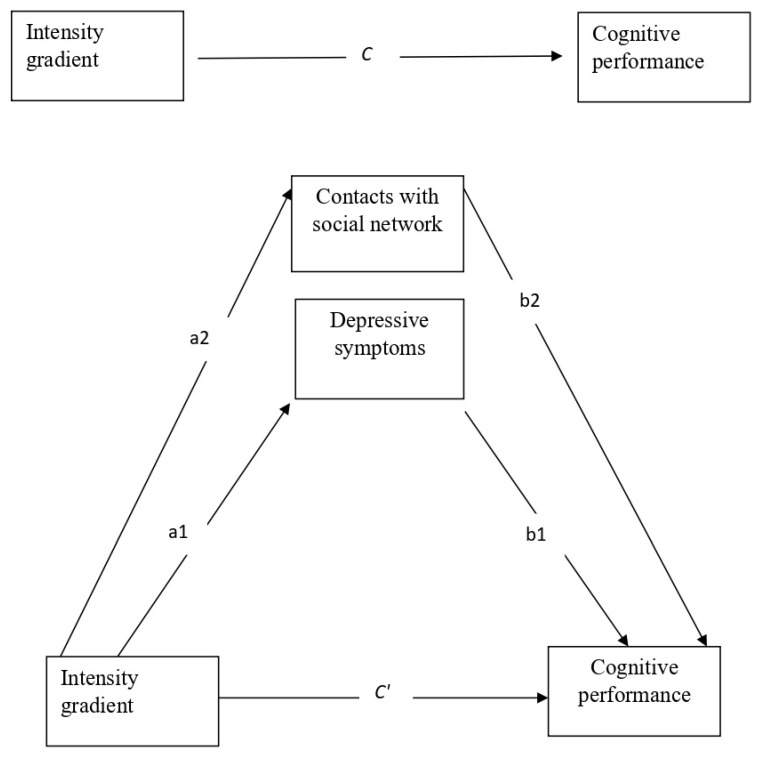
The main study model—a multiple mediator model depicting the direct effect without mediators (weight C) and with mediators (weight C′); and the indirect effects (sum of all a × b weights) of the intensity gradient via the two mediators (contact with the social network and depressive symptoms), controlling for the covariates.

We first analyzed descriptive statistics of means, standard deviations, and frequencies of the study variables. Next, we used Pearson correlations to examine the bivariate association between the study variables and the dependent variable of accelerometer-based physical activity. We then ran a multiple mediator analysis in which the two mediators (contacts frequency and depressive symptoms) were entered into the model. For this analysis we used the PROCESS model 4, which allows to simultaneously calculate the direct effect (weight c′, with mediators), total effect (c, without mediators) and indirect effect (a × b weights) of an independent variable on a dependent variable [65,66]. The multiple mediation model was examined by testing the significance of the mediating effects of the two mediators (a1 × b1, and a2 × b2) in the indirect link between the independent variable (the intensity gradient) and the dependent variable (cognitive performance), while controlling for covariates. Figure 1 presents the multiple mediator model that was examined in the main analysis.

We calculated the effects in this study through a bootstrapping set at 5000 samples. We calculated confidence intervals using this method by sorting the lowest to highest of these 5000 samples, resulting in a 95-percentile confidence interval. That is, if the number 0 is found within the range of this confidence interval, then the tested effect is non-significant. We ran the analyses using SPSS 25.0 with the PROCESS statistical program [66]. PROCESS reports estimated effects as unstandardized regression coefficients. 

## 5. Results

Table 1 shows the descriptive characteristics of the sample. It indicates that a over half of the participants were women (58.2%). Age ranged between 50 and 99 (Mean = 70.6, S.D = 8.9). About 71.5% had secondary education or below. On average the network size was 3.1 (S.D = 1.6). The respondents reported, on average, 2 chronic diseases (S.D = 1.5), the majority (75.2%) perceived their health as less than very good and about half (50.7%) reported severe or moderate limitations with doing activities due their health. The majority (91.1%) of the respondents reported that they were physically active, and their average score on intensity gradient (IG) was at a medium level with an average of −2.42 (range: −6 to −1). The average contact frequency with the social network was 6.3, which indicates that the respondents contacted their social network members between “several times a week” and “daily”. They also reported an average of 2.4 depressive symptoms (range: 0–12). As the cognitive performance indicator is based on standardized scores, its mean value is around zero, by design. 

Table 2 displays the outcomes of the Pearson correlations between the dependent variable (cognitive performance), the independent variable (intensity gradient), the mediators (depressive symptoms and contact frequency), and the covariates. It shows that respondents with a high intensity gradient (more active), and those who contacted their social network more often, also functioned better cognitively. More depressive symptoms, on the other hand, were associated with worse cognitive status. The analyses also showed that advanced age, more chronic diseases, and being limited with activities due to health were negatively correlated with cognitive performance, while educational level and a large social network were positively correlated. Cognitive performance was also better among respondents who reported engaging in physical activity and among those reporting better physical health. The results also showed significant links between self-reported physical activity and the accelerometer-assessed intensity gradient. The results also indicated that the study variables were not strongly related (r ≤ 0.42). This indicates that there are no grounds for possible multicollinearity between the variables in the current study.

Table 3 shows the results of the multiple mediation model (PROCESS model 4). The results indicated a significant total direct effect (path c; without mediators) of the intensity gradient on cognitive performance (B = 0.170, t = −2.69, *p* = 0.007, 95% CI = 0.046, 0.295; R^2^ = 0.210). Greater physical activity intensity (high intensity gradient score) was associated with better cognitive performance. The direct effect was also significant after taking the mediators into account (path c’; B = 0.19, t = 2.20, *p* = 0.028, 95% CI = 0.015, 0.264; R^2^ = 0.240). The results showed a significant indirect effect through contact frequency with the social network (B = 0.009, 95% CI = 0.009, 0.025) and through depressive symptoms (B = 0.022, 95% CI = 0.005, 0.046).

The results showed that the beta coefficient of the link between the intensity gradient and cognitive health declined from B = 0.170 without mediators to B = 0.139 after adding the mediators, which indicates partial mediation via social contacts and depressive symptoms. The results revealed that the intensity gradient was negatively associated with depressive symptoms (path a1) and this, in turn, was correlated with worse cognitive performance (paths b1). In addition, the intensity gradient was positively linked with more frequently contacting the social network (path a2) and this, in turn, was associated with better cognitive performance (path b2). 

Cognitive performance was also significantly associated with the covariates of gender, age, education, social network size, and reported physical activity. Advanced age and female gender were associated with lower cognitive health. However, higher education, large network size, and reported engagement in physical activity were associated with better cognitive performance. 

## 6. Discussion and Conclusions

The present study investigated the links between accelerometer-assessed physical activity and cognitive performance in later life, while also examining the mediators of this association. First, the results showed that adults aged 50+ years who engaged in more intense physical activity also had better cognitive performance. In addition, the association was mediated by social contacts and depressive symptoms. Individuals who engaged in intense physical activity contacted their social network more frequently and had fewer depressive symptoms, which in turn were linked to a better cognitive state.

Overall, our first hypothesis was supported, such that high intensity of accelerometer-assessed physical activity was associated with better cognition. Similar studies previously documented an association of accelerometer-assessed physical activity with cognition [14,16,17,23]. However, our findings go beyond existing research, which often focuses on specific categories of physical activity, by examining the novel metric of the intensity gradient. While such categories are related to overall activity level, the intensity gradient is different from overall activity levels and reflects the intensity distribution of the physical activity profile [18]. However, studies on the intensity gradient are largely limited to younger populations and do not focus on later life [19]. Our results expand the employment of the intensity gradient to adults aged 50+ and indicate that adults should be encouraged to engage in intense physical activities, which could benefit their cognitive performance. The results also showed a moderate connection between reported physical activity and the accelerometer-assessed intensity gradient. This result suggests a “true” association between accelerometer data and reported physical activities, as was also observed in previous studies [49]. Although we controlled for the reported physical activities in the final analysis, the accelerometer-assessed total physical activity had a significant contribution to cognitive performance, which may indicate that using the accelerometer-assessed intensity gradient is also relevant in relation to cognitive health. 

Additionally, the results showed that intense physical activity was correlated with cognitive health indirectly through more frequent contact with social network members. More intense physical activity could reflect going out more often, engaging in activities with other people, and meeting a larger range and number of individuals, while a more sedentary lifestyle could entail meeting fewer people. Frequent social contact might be related to having a more complex and diverse social environment, and previous studies also found it to be associated with better cognitive function in later life [67]. Such interactions could operate as a form of “cognitive exercise” which stimulates the mind and preserves cognitive functions in accordance with the “use it or lose it” hypothesis [24,25]. 

The third hypothesis was also confirmed. We found that accelerometer-assessed physical activity is not only directly linked with cognition, but that it can be related to cognitive health through a psychological avenue of lower depressive symptoms. Although, after adding this indirect link to the model, the direct association shrank (albeit remaining significant). This indicates that part of the direct association of physical activity intensity with cognitive health might be explained by mental health [23]. Engaging in intense physical activity could make adults feel less depressed and one of the effects of their improved mental health could be better cognitive function. Previous studies also found an association of depressive symptoms with cognitive function [22], and we go beyond such earlier findings by elucidating the role of depressive symptoms as mediators between physical activity and cognition. 

This study has several strengths, including the use of an accelerometer. Our study also had some limitations. First, the study sample focuses on only a subsample of participants in SHARE Wave 8 (855 panel participants) who took part in the accelerometer study. A second limitation is that due to the complexity of data collection of total physical activity using accelerometer, measurements are taken only from one body part (upper thigh), but the resulting inferences are applied to the whole body. An additional limitation concerns the possibility of reverse causation, given the cross-sectional nature of the current analyses due to the availability of accelerometer data for only one wave of SHARE. For example, it is also possible that adults in better cognitive state are also more physically active in their daily life. We thus emphasize that future studies should examine these results using longitudinal data when it will become available [2,16]. Furthermore, we included self-report health measures as covariates but did not include objective health measures, such as biomarkers or information from medical records, which are not currently available in the SHARE data. The PROCESS output includes only unstandardized coefficients, which does not allow us to compare the magnitude of effects. Future studies could use additional methods that allow examining the effect size. Additionally, the current study is based on data collected before the outbreak of the COVID-19 pandemic. However, during the pandemic some adults reported a decline in their physical activity [68]. Thus, future research should also examine the post-pandemic context of physical activity in later life.

In summary, the current study showed that more intense accelerometer-measured physical activity is directly related to better cognitive performance among European adults aged 50+ and is related indirectly via more frequent social contacts and lower depressive symptoms. With the rapid increase in the number of older adults around the world, our results have potential public health implications and can guide policy, services, and intervention programs. The findings suggest that brain health programs can benefit from including intense physical activities. Physical activity interventions for adults aged 50 and over, such as an exercise program with adjusted levels of intensity, may be related to better mental health, and to frequent contacts with network members. This, in turn, could be linked to good cognitive health. Such interventions should include a social component and encourage interactions between group members. 

## Figures and Tables

**Table 1:** Descriptive statistics of the study variables (N = 855).

Covariates		Valid N (%)	Mean (S.D)	Range
Gender	Men	357 (41.8)		
	Women	498 (58.2)		
Age			70.6 (8.9)	50–99
Education	Secondary education and below	606 (71.5)		
	Post-secondary education	241 (28.5)		
Social network size			3.1 (1.6)	0–7
Number of chronic diseases			2.0 (1.5)	0–9
Perceived health	Less than very good	643 (75.2)		
	Very good/excellent	212 (24.8)		
Limited with activities (GALI)	Limited	433 (50.7)		
	Not limited	421 (49.3)		
Physical activity	Never vigorous nor moderate physical activity	76 (8.9)		
	Other	779 (9–1.1)		
Independent variable				
Intensity gradient (IG)			−2.42 (0.43)	−6–−1
Mediators				
Depressive symptoms			2.42 (2.1)	0–12
Contacts with social network			6.29 (0.9)	1–7
Dependent variable				
Cognitive performance			0.15 (0.7)	−2.58–1.94

**Table 2:** Pearson correlations between study variables (N = 855).

	1.	2.	3.	4.	5.	6.	7.	8.	9.	10.	11.
Cognitive performance	1.00										
2. Intensity gradient	0.22 ***	1.00									
3. Depression	−0.20 ***	−0.21 ***	1.00								
4. Contacts with social network	0.15 ***	0.06	0.03	1.00							
5. Gender (Men)	0.05	0.14 ***	−0.19 ***	−0.04	1.00						
6. Age	−0.30 ***	−0.22 ***	0.04	0.08 *	0.11 **	1.00					
7. Education	0.28 ***	0.12 **	−0.10 **	0.20 ***	0.04	−0.06	1.00				
8. Perceived health	0.17 ***	0.18 ***	−0.25 ***	0.02	−0.02	−0.16 ***	0.12 **	1.00			
9. Social network size	0.21 ***	0.04	−0.01	0.37 ***	−0.12 ***	−0.03	0.16 ***	0.08 *	1.00		
10. Number of chronic diseases	−0.16 ***	−0.17 ***	0.30 ***	0.01	−0.02	0.24 ***	−0.11 **	−0.34 ***	0.01	1.00	
11. GALI	−0.11 **	−0.22 ***	0.35 ***	0.05	−0.10	0.10 **	−0.04	−0.39 ***	0.04	0.42 ***	1.00
12. Physical activity	0.18 ***	0.23 ***	−0.17 ***	0.03	0.03	−0.13 ***	0.04	0.11 **	0.06	−0.16 **	−0.16 ***

Notes: Value labels of categorical variables: gender (0 = female, 1= male); number of chronic diseases, perceived health (0 = less than very good, 1 = very good/excellent); Global Activity Limitation Indicator (GALI) (0 = not limited, 1 = limited); and physical activity (0 = never vigorous nor moderate physical activity, 1 = Active). * *p* < 0.05, ** *p* < 0.001, *** *p* < 0.001.

**Table 3:** Direct and indirect effect of intensity gradient on cognitive performance through depression and contacts with family (N = 855).

Direct Effects Intensity Gradient on Cognitive Performance Controlling Covariates–Without Mediators					Adj R^2^ (*p*. Value)
	B	95% CI		*p*. value	0.210 (<0.001)
Path C: Intensity gradient → cognitive performance	0.170	0.046	0.295	0.007	
Effects of Intensity gradient on mediators:					
Path a1: Intensity gradient → depressive symptoms	−0.444	−0.804	−0.084	0.015	
Path a2: Intensity gradient → contacts with social network	0.145	0.015	0.274	0.028	
Direct effects of Intensity gradient on cognitive performance controlling covariates–with mediators					
	B	95% CI		*p*. value	0.240 (<0.001)
Path C′: Intensity gradient → cognitive performance	0.139	0.015	0.264	0.028	
Path b1: Depressive symptoms → cognitive performance	−0.049	−0.073	−0.026	0.000	
Path b2: Contacts with social network → cognitive performance	0.063	0.006	0.119	0.027	
Covariates					
Gender	−0.105	−0.199	−0.010	0.029	
Age	−0.019	−0.024	−0.013	0.000	
Education	0.327	0.233	0.420	0.000	
Social network size	0.051	0.022	0.080	0.000	
Number of chronic diseases	−0.001	−0.032	0.030	0.934	
Perceived health	0.062	−0.047	0.173	0.262	
Limited with activities	0.018	−0.080	0.118	0.710	
Physical activity	0.220	0.010	0.430	0.039	
Indirect effect on cognitive performance via the mediators:					
	B	95% CI		SE	
Depressive symptoms (a1 × b1)	0.022	0.005	0.046	0.010	
Contacts with social network (a2 × b2)	0.009	0.009	0.025	0.006	

Notes: Value labels of categorical variables: gender (0 = female, 1= male); number of chronic diseases, perceived health (0 = less than very good, 1 = very good/excellent); limited with activities—GALI (0 = not limited, 1 = limited); and physical activity (0 = never vigorous nor moderate physical activity, 1 = Active).

## Data Availability

Publicly available datasets were analyzed in this study. This data can be found here: http://www.share-project.org/ (accessed on 1 January 2022).
